# Bayesian clustering and feature selection for cancer tissue samples

**DOI:** 10.1186/1471-2105-10-90

**Published:** 2009-03-18

**Authors:** Pekka Marttinen, Samuel Myllykangas, Jukka Corander

**Affiliations:** 1 Department of Mathematics and Statistics, University of Helsinki, FIN-00014, Finland; 2 Department of Pathology, Haartman Institute, University of Helsinki, FIN-00014, Finland; 3 HUSLAB, Helsinki University Central Hospital, University of Helsinki, FIN-00014, Finland; 4 Department of Mathematics, Abo Akademi University, FIN-20500, Turku, Finland

## Abstract

**Background:**

The versatility of DNA copy number amplifications for profiling and categorization of various tissue samples has been widely acknowledged in the biomedical literature. For instance, this type of measurement techniques provides possibilities for exploring sets of cancerous tissues to identify novel subtypes. The previously utilized statistical approaches to various kinds of analyses include traditional algorithmic techniques for clustering and dimension reduction, such as independent and principal component analyses, hierarchical clustering, as well as model-based clustering using maximum likelihood estimation for latent class models.

**Results:**

While purely algorithmic methods are usually easily applicable, their suboptimal performance and limitations in making formal inference have been thoroughly discussed in the statistical literature. Here we introduce a Bayesian model-based approach to simultaneous identification of underlying tissue groups and the informative amplifications. The model-based approach provides the possibility of using formal inference to determine the number of groups from the data, in contrast to the *ad hoc *methods often exploited for similar purposes. The model also automatically recognizes the chromosomal areas that are relevant for the clustering.

**Conclusion:**

Validatory analyses of simulated data and a large database of DNA copy number amplifications in human neoplasms are used to illustrate the potential of our approach. Our software implementation BASTA for performing Bayesian statistical tissue profiling is freely available for academic purposes at

## Background

Extensive research efforts have demonstrated the emergence and central role of DNA copy number amplifications in a wide variety of cancerous tissues [1-3]. Amplification multiplies the DNA copy number of a specific genomic region in a cancer cell and activates oncogenes by increasing their functionality by providing excess gene copies. Amplifications are clinically relevant genomic anomalies.

Amplification-activated oncogenes are frequently used as biomarkers of poor prognosis and, as amplified genes are attractive therapeutic targets, drugs inhibiting the function of amplified genes have been developed [4]. For example, Trastuzumab is a monoclonal antibody that inhibits the kinase activity of *ERBB2 *tyrosine kinase receptor, which is frequently overexpressed in breast cancer due to an amplification of the *ERBB2 *gene. Amplifications are relatively prevalent in different types of cancers and large amounts of genome-wide data are publicly available. Specific amplifications have been shown to associate with specific cancer types that share a similar etiological background. For example, cancers of the gastrointestinal tract clustered together according to their genome-wide amplification frequency profiles [1]. Similarly, using a machine learning approach, gastrointestinal tumors were shown to associate with amplifications at 2q32, 3q11.1-q29, 3p26-q29, 5p15.3-p11, 13q11-q34, 17q12-q21 and 17p13-q25 [2]. Due to their high prevalence, clinical value and the cell lineage-specificity, DNA copy number amplification patterns are feasible in characterizing cancer subtypes.

Various statistical approaches to profiling of tissue samples with respect to the patterns of gene amplification have been earlier exploited in the literature. Purely algorithmic techniques, such as independent and principal component analysis, as well as hierarchical and k-means clustering, have often been proposed for the purpose of extracting interesting and relevant patterns from high-dimensional and noisy data. Such methods are widely available in generic software packages and are typically fairly easily applicable by non-experts. However, despite their seemingly casual applicability, difficult methodological questions often arise in the context of such methods, of which the statistical uncertainty related to the derived solutions, and the choice of an appropriate number of clusters or dimensions are the most prominent ones. Therefore, an extensive statistical and computer scientific literature exists, where the advantages of model-based methods to solving pattern recognition problems compared to more *ad hoc *strategies in general are discussed [5,6].

Recently, [2,7] utilized a model-based clustering approach to statistical profiling of human cancers. In their method, clusters are inferred with maximum likelihood estimation utilizing the EM-algorithm and an appropriate number of clusters is chosen through cross-validation. From theoretical point of view the EM-algorithm is related to the Gibbs sampling Markov chain Monte Carlo (MCMC) algorithm (see e.g. [8]), as in both the algorithms the parameters for the different clusters are updated in an iterative fashion, based on the conditional distributions of the cluster labels for the samples. Although these types of algorithms are generally applied to model-fitting problems, the theoretical and practical limitations for situations involving a large number of underlying clusters are acknowledged in the statistical literature [8-10]. To avoid such problems, [2] performed their analyses separately for each chromosome. Although such an approach was relatively well motivated for the particular data set on human cancers analyzed by them, it would in general be statistically more coherent to consider all amplification data in a single joint framework. In particular, when tissue subgroups are associated with DNA copy number amplifications over multiple chromosomes, their reliable detection may require joint consideration of all data.

Here we introduce a Bayesian model-based approach to simultaneous identification of underlying tissue groups and informative DNA copy number amplifications. The model-based approach provides the possibility of using formal inference to determine the number of groups from the data, in contrast to the *ad hoc *methods often exploited for similar purposes. The model also automatically recognizes the chromosomal areas that are relevant for the clustering. The model is utilized in conjuction with a fast greedy stochastic algorithm, whose superior performance compared to an optimized version of Gibbs sampling MCMC algorithm is illustrated with a simulation study. A large number of simulated data sets are analyzed to determine the accuracy and sensitivity of the inferences derived using our method. We illustrate further the potential of the approach by analyzing jointly the large database of human neoplasms considered earlier by [2], who investigated each chromosome separately. We compare the performance of our method with that of a standard mixture modeling approach with no feature selection, and show for both the simulated and real data that the latter generally fails to discover biologically important patterns when the level of complexity in the data is moderate to high. Our software implementation BASTA for performing Bayesian statistical tissue profling is freely available for academic purposes at 

## Methods

### Bayesian clustering model for tissue samples

Here we consider a set of *n *tissue samples, which are profiled using DNA copy number amplifications, as described previously. The data are assumed to represent *d *binarized signals, where zeros and ones correspond to non-amplified and amplified chromosome bands, respectively. The observed binary vector for sample *i *is denoted by **x**_*i*_, *i *= 1,..., *n*, which takes values in the binary hypercube {0, 1}^*d*^. The set of *n *such binary vectors will be jointly denoted as **x**.

By modifying suitably the approach of [11] to amino acid sequence clustering, we obtain a statistical representation of the learning problem of simultaneously assigning the tissue samples into homogeneous subgroups and identifying informative chromosome sub-bands, as well as group-specific amplifications. These goals can be united by defining a family of probability models with a space of parameter configurations corresponding to the biological target. On one hand, this requires the identification of a suitable integer *k *and a corresponding clustering of the tissue samples into non-overlapping classes *s*_1_, *s*_2_,..., *s*_*k*_, which can be represented by a partition *S *of the data set. On the other hand, the model structure should capture a division of the sub-bands into biologically meaningful subgroups, which are here defined as noninformative and informative, depending on whether they provide information about the clustering. Furthermore, the model structure should identify for each cluster a subgroup of informative sub-bands amplified in the particular cluster. The considered model structure is analogous to that introduced by [11], apart from the fact that the amplification data is intrinsically binary, whereas [11] utilized a transformation of multinomial data vectors to develop a clustering model for binary quantities. Given the completely different biological purposes of the protein sequence analysis and pattern recognition for DNA copy number amplifications, we will re-formulate the original model here for the current context to provide better understanding for which type of signals the model aims to discover.

Biological relevance of the model configuration is ensured by imposing probabilistic assumptions on the observed binary data under the various specified categories. Firstly, when the sub-bands are such that the probability of observing them as amplified is invariant with respect to the underlying classes of samples, they are considered *noninformative*. That is, from the statistical perspective, such features of data contain no information for classifying tissue samples into relevant groups. Secondly, when the probability of observing a particular sub-band as amplified differs over the classes, it is considered *informative *for the present purposes. Given the set of noninformative sub-bands, the latter group equals simply the complement set among all *d *data features. Thirdly, we allow the model to specify subsets of informative sub-bands which are group-specific, such that the probability of observing a particular sub-band as amplified is very high in a specific class of tissue samples, whereas it is relatively low outside this class. We denote by D⊆{1,...,d} all the informative sub-bands, and by $T=(T_{1},...,T_{k})$ the subsets of D corresponding to the group-specific amplifications.

As the categorization of the amplifications into the three specific types is statistically intimately connected to the clustering *S *of the samples, it is instructive to define the model structure as a triplet M=(S,D,T). Any instance of such a triplet can be combined with a predictive probability distribution for the observed data analogously to the learning rules developed from generalized exchangeability in [12] in a different biological context. In particular, the work by [12] shows how an unsupervised Bayesian classification scheme arises for gene marker data under a random urn model and certain conditions regarding the stochastic process by which the data are assumed to have been generated. It should be noted that, unlike in the present context, in their work all observed features are considered relevant for classification by the model. Let the model space ℳ contain all the triplets of the type specified above. The predictive Bayesian learning then proceeds by converting the joint distribution

(1) *p *(**x**, *M*) = *p *(**x**|*M*) *p *(*M*),

where *p *(*M*) represents the *a priori *uncertainty about the model structure, into the posterior distribution

(2) $$p\left(M|x\right)=\frac{p\left(x|M\right)p\left(M\right)}{\sum_{M\in ℳ}p\left(x|M\right)p\left(M\right)},$$

which is utilized as the basis for identifying model structures that efficiently and parsimoniously capture features of the data in an information-theoretic manner. Our biological target is to identify the model structure *M *that maximizes the posterior probability (2), and to find representations of the amplification informativeness over the considered chromosomal bands using the features of *M*. In concrete terms, our learning algorithm described below alternates between the two operators targeting to maximize (2) with respect to either the partition or the configurations of the sub-bands. The maximization of either one of these structural parameters is always performed conditional on the current value of the other parameter. To concretize the predictive component *p *(**x**|*M*) of the learning model, we employ exchangeability assumptions concerning the observation of particular amplifications under the elements of the triplet M=(S,D,T) analogously to [11]. These lead to the following predictive likelihood

(3) $$\begin{array}{c} p(x|M)=\int_{\Theta }\left(\prod_{c=1}^{k}\prod_{j\in D\cap T_{c}}\prod_{l=0}^{1}p_{cjl}^{n_{cjl}}\right)\left(\prod_{c=1}^{k}\prod_{j\in D\backslash T_{c}}\prod_{t=0}^{1}q_{cjl}^{n_{cjl}}\right)\\ {}*\left(\prod_{j\notin D}\prod_{l=0}^{1}w_{jl}^{n_{jl}}\right)p(\theta )d\theta , \end{array}$$

which involves three distinct types of conditional probabilities. Firstly, *p*_*cjl*_, *c *= 1,..., *k*, *j *∈ $j\in D\cap T_{c},l=1$, represents the probability that sub-band *j *is amplified among the tissues allocated in class *c*, given that *j *is a group-specific amplification for class *c*. Similarly, *p*_*cjl*_, *l *= 0, represents the probability of the absence of amplification. Secondly, *q*_*cjl*_, *c *= 1,..., *k*, *j *∈ $j\in D\backslash T_{c},l=0,1$, are the corresponding probabilities for the sub-bands that are not group-specific for the class *c*. Finally, *w*_*jl*_, j∉D, *l *= 1, is the probability of an amplification in any noninformative sub-band, and *w*_*jl*_, *l *= 0, the probability of absence of amplification, which both are equal for all classes of tissue samples. In (3), the parameter *θ *represents jointly all probabilities *p*_*cjl*_, *q*_*cjl *_and *w*_*jl*_. The observed counts of presence and absence of an amplification are given by the sufficient statistics *n*_*cjl*_, *n*_*jl*_, *c *= 1,..., *k*, *j *= 1,..., *d*, *l *= 0, 1.

Under the following conjugate Beta priors (see e.g. [13]) for the probabilities in (3):

(4) $$\begin{array}{l} p_{cj1}\sim Beta\left(\lambda_{1},\lambda_{0}\right),p_{cj0}=1-p_{cj1},\\ q_{cj1}\sim Beta\left(\mu_{1},\mu_{0}\right),q_{cj0}=1-q_{cj1},\\ w_{j1}\sim Beta\left(\nu_{1},\nu_{0}\right),w_{j0}=1-w_{j1}, \end{array}$$

the predictive probability is available analytically as

(5) $$\begin{array}{c} p\left(x|M\right)=\prod_{i=1}^{k}\prod_{j\in D\cap T_{i}}\frac{\Gamma \left(\sum_{l=0}^{1}\lambda_{l}\right)}{\Gamma \left(\sum_{l=0}^{1}\lambda_{l}+n_{ijl}\right)}\prod_{l=0}^{1}\frac{\Gamma \left(\lambda_{l}+n_{ijl}\right)}{\Gamma \left(\lambda_{l}\right)}\\ {}*\prod_{i=1}^{k}\prod_{j\in D\setminus T_{i}}\frac{\Gamma \left(\sum_{l=0}^{1}\mu_{l}\right)}{\Gamma \left(\sum_{l=0}^{1}\mu_{l}+n_{ijl}\right)}\prod_{l=0}^{1}\frac{\Gamma \left(\mu_{l}+n_{ijl}\right)}{\Gamma \left(\mu_{l}\right)}\\ {}*\prod_{j\notin D}\frac{\Gamma \left(\sum_{l=0}^{1}\nu_{l}\right)}{\Gamma \left(\sum_{l=0}^{1}\nu_{l}+n_{jl}\right)}\prod_{l=0}^{1}\frac{\Gamma \left(\nu_{l}+n_{jl}\right)}{\Gamma \left(\nu_{l}\right)}. \end{array}$$

Here *λ*_*l*_, *μ*_*l*_, *ν*_*l*_, *l *= 0, 1 are hyperparameters whose values represent beliefs concerning the expectation of the observable amplification prevalence under the three distinct categories.

The statistical learning model still requires the assignment of a prior distribution to values of the triplets (S,D,T) to describe the uncertainty about the presence of homogeneous hidden subgroups of tissue samples and the identities of the informative amplifications. This distribution is determined by

(6) p(S,D,T)=p(S)p(T,D|S),

where we set the probability *p *(*S*) as

(7) *p*(*S*) = C* *I *(|*S*| ≤ *K*),

where *C *is a constant, and *I *(|*S*| ≤ *K*) is an indicator function which equals unity, if the partition *S *has at most *K *clusters, and zero otherwise. *K *denotes here a user-specified upper bound for the number of clusters, considered to be suitable for the problem at hand. Thus, (7) assigns equal probability to all partitions which have at most *K *clusters. The conditional prior for the categorization of the amplifications is then specified according to

(8) $$p\left(T,D|S\right)\propto \xi^{\sum_{c=1}^{k}\left|D\cap T_{c}\right|}{\left(1-\xi \right)}^{\sum_{c=1}^{k}\left|D\backslash T_{c}\right|},$$

where *ξ *is the probability that a particular amplification is group-specific for some class, and |·| denotes the cardinality of a set. The prior can be derived by considering the status of the informative amplifications in an arbitrary class. Let *ξ *be the probability of a Bernoulli event that an amplification is group-specific in some class, and thus, (8) is the product probability of such Bernoulli random variables over all classes and the informative amplications. Such a prior is therefore characterized by a penalty for complex models with an increasing number of classes and informative amplifications.

### Bayesian model learning algorithm

Here we provide brief details for a stochastic search algorithm that is used to identify the optimal Bayesian solution to the classification problem, *i.e*. the triplet (S,D,T) maximizing the posterior probability

(9) $$\hat{M}=\arg\underset{M}{\max}p\left(M|x\right).$$

The various intelligent search operators used in the algorithm are inspired by the novel non-reversible Metropolis-Hastings approach to Bayesian model learning introduced in [14], modifications of which have been applied by [11] and by [15], the latter work considering semi-supervised classification of fish samples based on gene marker data. The non-reversible algorithm of [14] can be considered as a generic Bayesian learning tool for models for which predictive likelihoods can be analytically calculated given any particular configuration of the structural layer of a model.

As in [11], the model optimization consists here of two primary elements, the search for an optimal partition and the optimal categorization of the sub-bands into the three groups (noninformative, informative, group-specific). Our algorithm proceeds by repeatedly applying a variety of transition operators to the current state corresponding to a particular configuration of *M*. The changes that lead to an improvement in the posterior probability are accepted. In particular, after each proposed change to the partition *S*, the optimal groups of informative (D) sub-bands and group-specific (T) amplifications are identified, and using these, the putative partition is evaluated in terms of *p *(**x**|*M*) *p *(*M*). The search for $\hat{M}$ is terminated when no improvement in the model is accessible. The different search operators utilized to search the partition space are as follows:

1. Move a tissue sample from one class to another.

2. Join two classes.

3. Split a class using complete linkage clustering (see e.g. [16]) with some specified distance measure.

Due to the complexity of the posterior distribution with respect to the model space topology, entirely local perturbations to the model structure, such as step 1 above and those employed in generic Gibbs sampler algorithms [8], would easily converge to a local maximum of the posterior. Therefore, it is of importance to utilize intelligent operators enabling successful large jumps in the search space, as in the last step. The variety of Hamming distance measures that we utilize in the splitting step are analogous to those considered in [11]. To facilitate the identification of the global optimum it is advisable to perform the search multiple times using alternative initial configurations. In cases where the algorithm identifies separate maxima on different runs, the obtained solutions can be coherently compared using the predictive likelihoods and prior probabilities *p *(**x**|*M*) *p *(*M*).

To actually operationalize the model learning, it is still necessary to explicitly specify the prior hyperparameters in (8) and (4). As demonstrated in [11], the stochastic partition model with feature filtering is not sensitive to the specific choices of the hyperparameter values, given that they are within a biologically meaningful range. The value for the prior probability *ξ *that an arbitrary informative sub-band contains a group-specific amplification for some class is needed in the definition of the joint prior (6) for the qualitative layer of the model. In our default software implementation *ξ *is chosen to be equal to 0.01. This choice prevents efficiently the emergence of spurious singleton classes, when the data contains a limited amount of information in terms of the number of informative chromosomal bands. As the amount of data increases, the effect of this prior becomes negligible, and in general, the exact value of *ξ *is of no crucial importance.

We also need to specify the prior distributions for the model parameters *p*_*ij*1_, *q*_*ij*1_, and *w*_*j*1_, that define probabilities for an amplification being present when the corresponding sub-band is categorized as noninformative or informative (possibly group-specific). Here we apply the findings of [11], to scale the hyperparameters in an automatic fashion with respect to the amount of information in any given data set. The priors are specified according to:

$$\begin{array}{l} p_{ij1}\sim Beta\left(\alpha \phi_{i},\left(1-\alpha \right)\phi_{i}\right)\\ q_{ij1}\sim Beta\left(f*\phi_{i},\left(1-f\right)*\phi_{i}\right)\\ w_{j1}\sim Beta\left(f*n,\left(1-f\right)*n\right). \end{array}$$

where *n *is the total number of samples and *f *is the overall frequency of observed amplifications in the data. The term *φ*_*i *_is a scaling factor that depends on the number of samples assigned in class *i*, such that

*φ*_*i *_= max {|*s*_*i*_|, 10},

where |*s*_*i*_| is the cardinality of class *i*. The hyperparameter *α *∈ [0, 1] determines the expected number of amplifications at a specific sub-band to be observed among the samples in a class, if the particular amplification is group-specific according to the model. Thus, the value given to *α *should be fairly high to restrict the attention to strong signals of group-specificity. In our default software implementation we have used the value *α *= .95, as this choice allows for some flexibility for the observed counts of zeros and ones, while preventing contradictory behavior of the model. However, in the validatory experiments reported below a range of different values of *α *was used to assess the sensitivity of the model with respect to this hyperparameter.

### Assessing model uncertainty

To provide a statistical measure for the model certainty in the vicinity of the estimated posterior mode, and to detect hot-spots over the considered chromosomal areas, where the amplifications yield an indication of being group-specific, or just informative, against being unrelated to the presence of heterogeneity among the tissue samples, we use posterior odds, which in the current situation allow conveniently the comparison of the relative plausibilities of any two models. Posterior odds for model *M*_1 _compared to *M*_2 _is given by

(10) $$R_{12}=\frac{p\left(x|M_{1}\right)p\left(M_{1}\right)}{p\left(x|M_{2}\right)p\left(M_{2}\right)}.$$

With a sufficient amount of data the ratio of the marginal likelihoods (the first part of the ratio in (10)) dominates the odds and consequently, the odds will be closely related to Bayes factors [17], especially under the utilized fairly non-informative prior choices for the models. Posterior uncertainty related to the assignment of any particular tissue sample *i *to the class in the optimal solution can now be characterized using (10).

Assume that in the optimal model $\hat{M}=M_{1}=\left(S_{1},D_{1},T_{1}\right)$, the tissue sample *i *belongs to the estimated class *c *and we seek to compare this to an alternative classification where the classes are kept otherwise similar, except that the sample *i *is re-assigned to another class, say c*. Let the new partition be denoted by *S*_2_. A competing model $M_{2}=\left(S_{2},D_{2},T_{2}\right)$ may thus be obtained by optimizing $D_{2}$ and $T_{2}$ with respect to the partition $S_{2}$. In this manner, the posterior odds (10) in favor of *M*_1_, and against *M*_2_, can be used as a measure of plausibility of the assignment of the samples into any of the alternative classes, which provides a statistical measure for the strength of the evidence for the estimated optimum solution. Notice that conditional posterior distribution over the alternative assignments of the tissue sample may also be easily obtained through a normalization of the terms involved in (10) by summing over the classes. To explore the evidence in the data over the considered chromosomal bands in order to screen for amplification hot-spots, posterior odds can be analogously computed sub-band-wise. In this case, a natural comparison is between the model claiming a particular sub-band to be informative, *e.g*. group-specific, against the alternative hypothesis that the sub-band is noninformative. Thereby, the values of the posterior odds can be used to screen for interesting areas over the chromosomes. These values, as well as the above mentioned types of posterior odds are automatically provided by our software implementation.

### Real database of DNA copy number amplifications

The collection of 4590 DNA copy number amplifications in human neoplasms was obtained from [4]. These data were collected from 838 publications that report results of comparative genomic hybridization experiments on cancer samples. The data are reported in chromosome sub-band resolution (*d *= 393). Amplifications are recorded in binary vectors where 0 and 1 denote non-amplified and amplified chromosome sub-bands, respectively. There are 73 different neoplasm types in the database.

### Simulation design for validatory experiments

To investigate the performance of the model discussed above, we generated a large number of synthetic data sets and analyzed them using the described learning algorithm.

Two basic simulation scenarios were used, with 30 and 50 underlying clusters of samples, respectively. For each scenario, three ranges of cluster sizes were considered, such that the number of samples in a cluster was distributed according to one of the following distributions: Uniform(5,20), Uniform(10,25), Uniform(15,30). For each of these six combinations of the number of clusters and their size distribution, 20 data sets were generated under each of three different random levels of noise in the data (see below). The parameter configuration used to generate a single data set was determined as follows. Firstly, the number of group-specific amplifications was sampled for each cluster from Uniform(5,8) distribution. To mimic the properties of real amplification data, the total number of binary attributes included was set to 393. The average total number of group-specific amplifications represented thus a bit over 50% of all attributes for the case with 30 clusters. For each cluster and amplification, the group-specific probability of observing an amplification was independently simulated from the Uniform(.8,1) distribution. To regulate the amount of noise in the data, we chose the probability of observing a group-specific amplification outside its group to be at one of three distinct levels, .01, .05, .1, independently for all clusters and group-specific amplifications. Out of the remaining attributes, 25% were randomly chosen as informative, and their generating probabilities were sampled from the Uniform(0,1) distribution independently for each cluster and attribute. The other 75% of the remaining attributes were chosen to represent noise, such that the generating probability was for each attribute sampled from the Uniform(0,1) distribution, and the same value was used for each cluster. Given the realizations of cluster size variables and of the generating probabilities for each cluster and attribute, a binary data set was sampled using them. An average data set with 30 clusters would thus have 375–675 observed samples under the three different distributions on cluster sizes. An analogous simulation scheme was used for the case with 50 clusters. For these data sets, the average proportion of noise attributes decreased to around 10%, because a considerably larger part of the 393 amplifications were modeled as group-specific. In total, there were thus 18 distinct sampling configurations, under each of which 20 parameter realizations and corresponding data sets were simulated, leading to 360 data sets.

Each simulated data set was analyzed using four distinct values of the hyperparameter *α*, equal to .875, .9, .95, .975, respectively, to investigate the sensitivity of the inferences with respect to the choice of the prior specification. Due to computational restrictions, stochastic optimization of the model was done only once for each data set. Thus, the results may be considered as somewhat conservative with respect to the performance, because the learning algorithm would be applied multiple times in any real data analysis situation, which increases the chance of identifying the globally posterior optimal model structure. In the stochastic optimization we used the upper limit for number of clusters *K *equal to 50 (70), when the number of generated clusters was 30 (50). For comparison, each data set was also analyzed using an alternative method based on clustering without feature selection (for details see the next subsection).

### Alternative methods

An extensive methodological literature exists for unsupervised clustering and feature selection. However, a majority of the articles in this area are concerned with the analysis of continuous data, often based on the use of Gaussian mixture models combined with the EM-algorithm for learning, e.g. [18,19]. Several interesting alternative Bayesian approaches to clustering discrete data with naive Bayes -type models and various generalizations of them exist, e.g. [20-24]. However, for most such methods, software implementations are not publicly available. Restricted by the availability of suitable software, we performed comparative analyses of the real amplification data using the EM-algorithm based latent class mixture modeling method implemented in AutoClass software [20] and the stochastic partition model implemented in BAPS software, e.g. [12] or [25]. Due to the very large number of performed analyses, we used only BAPS software in the comparison for the simulated data. To test an algorithmic alternative method, we considered the standard k-means algorithm, see for example [16]. As k-means does not provide a coherent way for estimating the number of clusters, the number of clusters was specified in this analysis to be the same as the number of clusters inferred by the method described above. Although the beta-binomial clustering model arising under the theory developed in [12], and the latent class mixture model for binary data discussed in [20] are conceptually derived under different theoretical frameworks, the resulting likelihood expressions are rather similar. Thus, it is expected that the two methods will yield similar inferences, if the differences in the computational strategies adopted in the methods do not dominate for a particular analysis. The beta-binomial clustering model specifies the likelihood of the data **x **as

$$p\left(x|M\right)=\int_{\Theta }\prod_{c=1}^{k}\prod_{j=1}^{d}\prod_{l=0}^{1}p_{cjl}^{n_{cjl}}p\left(\theta \right)d\theta ,$$

including now only one type of parameters *p*_*cjl*_, with a noninformative Jeffreys' prior (see e.g. [26]):

*p*_*cj*1 _~ *Beta *(1/2, 1/2), *p*_*cj*0 _= 1 - *p*_*cj*1_.

These specifications lead to the following form of the marginal likelihood:

$$p\left(x|M\right)=\prod_{i=1}^{k}\prod_{j=1}^{d}\frac{1}{\Gamma \left(1+\sum_{l=0}^{1}n_{ijl}\right)}\prod_{l=0}^{1}\frac{\Gamma \left(1/2+n_{ijl}\right)}{\Gamma \left(1/2\right)}.$$

Thus, in this simpler model the features are not divided into informative and noninformative, and further, no group-specific features are specified.

To illustate the potential of the stochastic greedy search algorithm for learning of complex models, we perform also a comparison with an optimized version of the Gibbs sampling algorithm. Both algorithms are used to cluster a group of artificial data sets, in order to compare their performances in solving an unsupervised classification task. The optimization refers here to the use of analytical integration to derive an expression for the conditional posterior probabilities of cluster labels for data items, where the parameters of class-conditional distributions are integrated out. In the standard version of the Gibbs sampling algorithm this integration is typically performed via additional simulation steps, where values for the parameters of the class-conditional distributions are generated, which increases the computational complexity and makes the algorithm less efficient.

The Gibbs sampling algorithm used in the comparison can be described as follows. Let the state of the Markov chain consist of *c*_*i*_, *i *= 1,..., *n*, the cluster labels for the data items, i.e. *c*_*i *_∈ {1,..., *K *} for all *i*, where *K *is the specified upper bound for the number of clusters. The cluster labels have no meaning beyond specifying which data items belong to the same cluster. Let *c*_-*i *_denote the cluster labels of other data items except *i*. The conditional distribution for the cluster label *c*_*i *_can be written as

(11) *P*(*c*_*i *_= *c|c*_-*i*_, **x**) ∝ *P*(**x**|*c*_*i *_= *c, c*_-*i*_) * *P*(*c*_*i *_= *c|c*_-*i*_),

where *P *(**x**|*c*_*i *_= *c*, *c*_-*i*_) is the marginal likelihood of the data conditional on the partition specified by the cluster labels, and *P *(*c*_*i *_= *c|c*_-*i*_) is the prior distribution for *c*_*i*_, conditional on the cluster labels of the other data items. By assuming that all partitions are *a priori *equally likely, it follows that

(12) *P*(*c*_*i *_= *c|c*_-*i*_) = *D*,

where *D *is a constant. Thus, the probabilities are the same for all clusters which are represented in *c*_-*i*_, and, in addition, one arbitrarily chosen empty cluster, while the probability of the rest of the empty clusters is zero (notice that assigning the data item to any of the empty clusters yields the same partition). Then, the Gibbs sampling algorithm simply consists of iterations, each of which updates in a random order the cluster labels for the data items, drawing the new values from the conditional distributions (11). Notice that the specified algorithm can be considered as a standard algorithm for sampling from Dirichlet process mixture models [27], apart from the different prior specification for the cluster labels.

To illustrate how the presented stochastic greedy optimization and the Gibbs sampling MCMC strategies behave in practice, we employ a series of artificial data sets. We analyze each such data with both the described algorithms. Due to computational simplicity, we employ in this comparison the algorithms with the simple beta-binomial model described above. Because in this comparison both algorithms are used to optimize the structure of the same model, the results can be considered illustrative also for the relative time complexities in the case where the feature selection is included in the learning problem. Both algorithms are executed twice, by starting the algorithms from two different initial setups. In the first initial setup all data items are assigned into a single cluster. For the second setup, the complete linkage clustering algorithm is utilized to create *k*_0 _+ 10 initial clusters, where *k*_0 _is the correct underlying number of clusters. The number of iterations in the MCMC is specified to be 1,000. We consider data sets of seven different degrees of complexity. The simplest data sets consist of 15 clusters, whereafter the number of clusters is increased up to 45 clusters using an equal spacing of 5 clusters. For each data set size, we generate five different data sets. The numbers of the data items are drawn independently for the different clusters from a uniform distribution over the set {5, 6,..., 20}. Each data item is characterized by a binary vector *x*^*i *^∈ {0, 1}^*d*^, where we use value *d *= 35 for the length of the vector. We draw the frequency parameters *θ*_*ij *_for different clusters *i *and features *j *independently from

$$\theta_{ij}\sim Beta\left(\frac{1}{2},\frac{1}{2}\right),$$

thus following the assumptions of the simple beta-binomial model. The value *d *= 35 was selected because in preliminary experiments we found it to be suitable for the comparison. With higher values (e.g. > 50) both the algorithms were able to find exactly the correct partition in a short time, while with lower values (e.g. < 20) the data was not informative enough for a proper learning of the partition, and consequently both algorithms, especially the Gibbs sampler, ended up far away from the optimal model. For reporting the results, we record for each run of the greedy algorithm the running time and the marginal likelihood of the model with the highest value. Because the total running time of the MCMC algorithm is largely determined by the pre-specified number of iterations, it provides an unsatisfactory basis for the comparison of the efficiencies of the algorithms. A more reasonable estimate of the running time of the MCMC algorithm is obtained by recording the time to reach a model with an equal or higher marginal likelihood than the highest value found by the greedy algorithm. If the MCMC never reaches a model with such a value during the 1000 iterations, we record the time needed to reach the model with the highest value in that run. To compare the goodness of the solutions found by the alternative algorithms, we select for both algorithms the model with the maximum marginal likelihood found in the two separate runs. We then use Bayes factors to compare these top-scoring models (notice that for the simple beta-binomial model the Bayes factor is equal to the posterior odds described earlier).

## Results and discussion

### Comparison of algorithms with synthetic data

First, we present results from the validatory simulation experiments, and thereafter, comparison of the stochastic optimization and Gibbs sampling algorithms is shown. The results from the validatory analyses are summarized in Additional file 1. Exact results from the alternative clustering method without feature selection are not shown in Additional file 1, because they are uniformly inferior compared to the method proposed here. On average, the alternative method detected only 6–7 clusters and the adjusted Rand Index of the resulting partition was at best approximately 50% of the corresponding value for the proposed method. In most cases, the alternative method yielded an adjusted Rand Index value that was in the range of 1–10% of the corresponding value reported in Additional file 1. Thus, without feature selection, mixture models are not expected to perform feasibly for pattern recognition of this type of data.

Additional file 1 reveals that the penalties for increasing model complexity included in the adopted Bayesian formulation are highly efficient, as there is no tendency to overestimate the number of clusters. Comparison of the four distinct values of hyperparameter *α *highlights that there exists a trade-off between the accuracy in the inferences about the number of clusters and about discovering the group-specific amplifications. Increasing the value of *α *brings in general the average number of inferred clusters closer to the generating model setup, however, simultaneously, there is a slight decrease in the proportion of correctly inferred group-specific amplifications. This is entirely expected, as a value of *α *closer to unity favors a very small fraction of zeroes to be observed for attributes that are considered by the model to be group-specific. However, the magnitude of differences between the different values of *α *suggests that the inferences are in general not very sensitive to the exact value chosen, as long as it is sufficiently large. Therefore, our default implementation with *α *= .95 would seem appropriate.

Values in Additional file 1 reflect well the effect of the level of noise present in the generated data. When the proportion of uniformative attributes is small (around 10% when *k *= 50), the inferences are very accurate for all levels of noise, i.e. the level of the presence of group-specific amplifications outside their groups. When the amount of uninformative attributes is very large (*k *= 30), the accuracy of inferences depends on the cluster sizes. Under such circumstances, it is not expected that a model may reliably estimate underlying generating probabilities for a very small cluster with size in the range 5–10 samples, unless the probabilities are either close to zero or unity. Even for the noisiest data sets, the estimation accuracy is remarkably high when cluster sizes are in the range 15–30.

**Figure 1:**
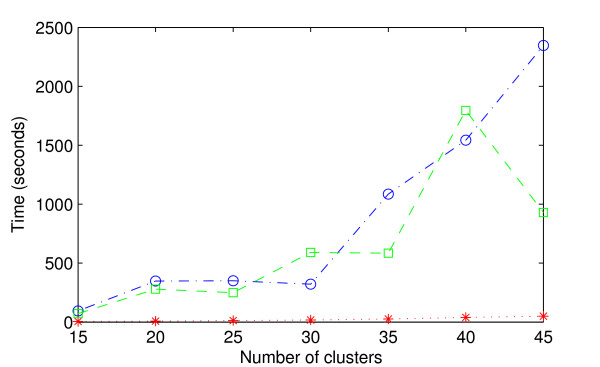
**Comparison of running times of the algorithms**. Results of the comparison of the running times of alternative algorithms (MCMC Gibbs sampling, greedy algorithm) on artificial data sets using the basic beta-binomial model. The x-axis shows the complexity level of the data set in terms of the number of clusters and the y-axis shows the average time for five data sets at each level in seconds. Because the times taken by the two versions of the greedy algorithm were roughly equal, they are represented by just one curve (red asterisks). The MCMC initialized by a single cluster is represented by a green square. The MCMC initialized with the correct number plus additional 10 clusters is represented by a blue circle. The times recorded for the MCMC correspond to the times when they reached a model with equal or higher marginal likelihood than the highest value found by the greedy algorithm. If the MCMC never found such a model, the recorded time is the time taken by the MCMC to reach the model with the highest marginal likelihood in that run.

**Figure 2:**
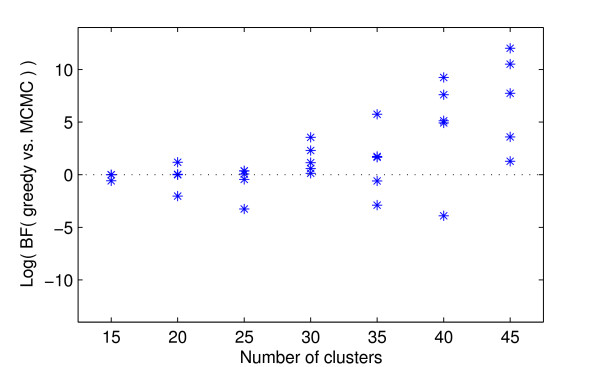
**Comparison of models found by the alternative algorithms**. Logarithm of the Bayes factor of *M*_1 _vs. *M*_2_, where *M*_1 _is the model with the highest marginal likelihood value found in the two runs of the greedy algorithm and *M*_2 _is the corresponding model obtained from the MCMC runs. The Bayes factor is shown for each analyzed synthetic data set. Thus, five values are shown for each data set size.

The results of the comparison of the two alternative algorithms, Gibbs sampling and the suggested stochastic greedy optimization, to learn the optimal partition for artificial data are shown in Figures 1 and 2. Figure 1 shows the average running times of the different algorithms. The results of the comparison of the optimal models found by the two algorithms are shown in Figure 2.

As can be seen from Figure 1, the running time of the MCMC algorithm increases rapidly as the complexity of the data set increases. In contrast, the greedy algorithm is considerably faster for all the data set sizes. For example, the execution times are approximately 60 times longer for the MCMC algorithm started from *k*_0 _+ 10 as compared to the greedy search, when the most complex data sets are considered. A first look to Figure 1 would surprisingly suggest that the MCMC started from a single cluster is performing faster than the MCMC initialized with *k*_0 _+ 10 clusters. A closer inspection (exact results not given here) shows, however, that this is not the case. Recall that the time shown in Figure 1 is the time required to reach a model with an equal value to that found by the greedy algorithm or, if no such model is found, a time required to reach the model with the highest marginal likelihood value in that run. As can be seen from Figure 2, neither of the MCMC runs found a model with equal predictive value to that found by the greedy algorithm for the data sets with *k*_0 _= 45. Moreover, for these data sets, the MCMC started with a single cluster never found a model of equal or higher value than the MCMC started with *k*_0 _+ 10 clusters. The surprisingly low running times (with *k*_0 _= 45) in Figure 1 for the MCMC started with one cluster are therefore explained by the fact the algorithm got faster stuck to a local mode in the model space than the MCMC started with *k*_0 _+ 10 initial clusters.

### DNA copy number amplification data

The DNA copy number amplification data described previously were analyzed using the default implementation of our method in the BASTA software. In particular, four runs of the estimation algorithm were performed using the user-specified upper bound *K *for the number of clusters equal to 250, 275, 300 and 325. For the first two runs the algorithm converged to a solution with number of clusters equal to the initial number of clusters *K*, indicating that the posterior optimum contains presumably a larger number of underlying tissue groups. In the latter two runs, the algorithm converged to exactly the same solution, consisting of 291 clusters of samples.

**Figure 3:**
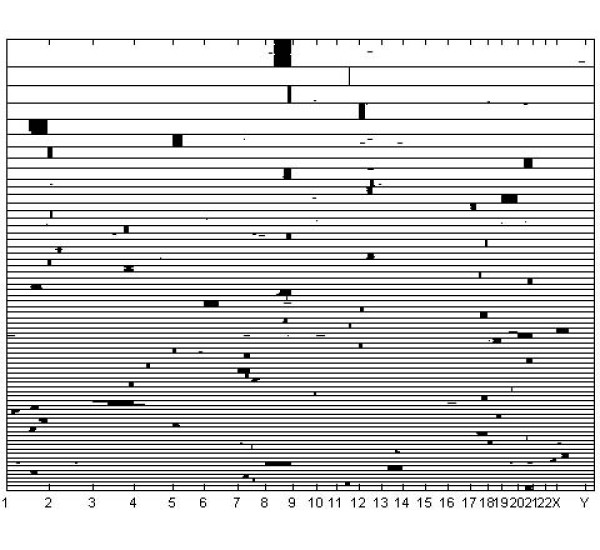
**Large clusters for the cancer data**. Inferred large clusters (20 or more samples in a cluster) for the DNA copy number amplification data. The samples on y-axis are ordered according to the inferred clustering, and the clusters are ordered starting with the largest cluster on top. The clusters are separated by horizontal black lines. x-axis positions correspond to amplification sub-bands, and amplified sub-bands are drawn in black for each sample. The tick marks on x-axis show the boundaries of chromosomes.

**Figure 4:**
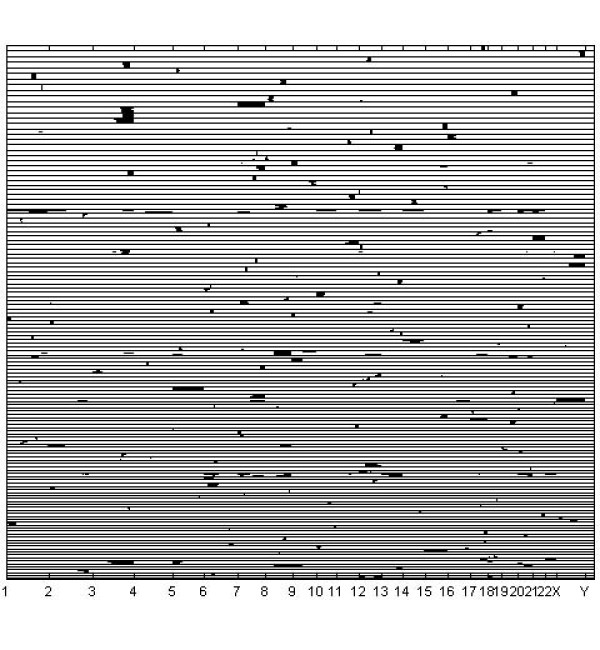
**Medium-sized clusters for the cancer data**. Inferred medium-sized (10–19 samples in a cluster) clusters for the DNA copy number amplification data.

**Figure 5:**
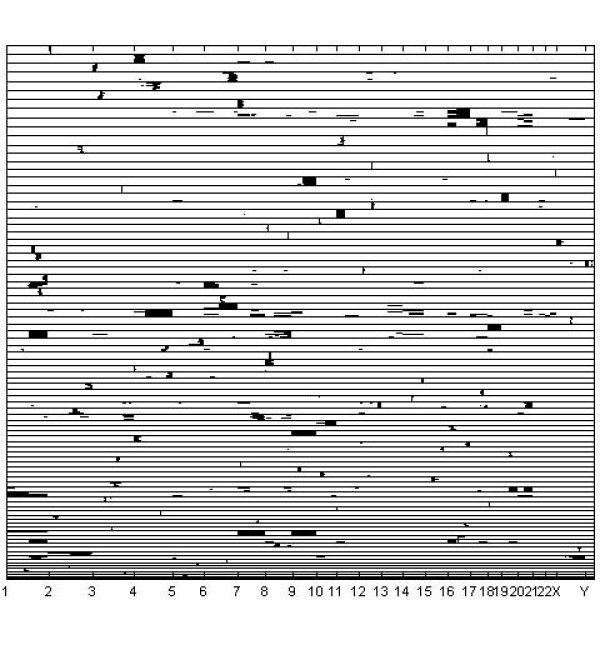
**Small clusters for the cancer data**. Inferred small clusters (size less than 10) for the DNA copy number amplification data.

**Figure 6:**
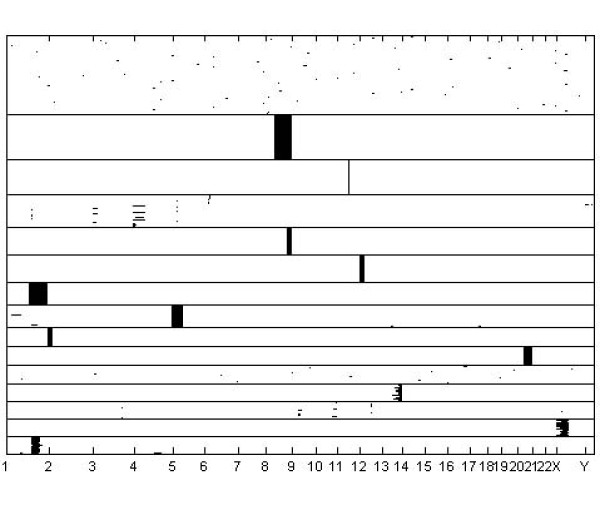
**K-means clustering results**. Fifteen largest clusters for the DNA copy number amplification data, obtained from a k-means analysis with the number of clusters set to 291.

A graphical image of the optimal clustering is shown in Figures 3, 4 and 5, such that Figure 3 contains clusters with size ≥ 20, Figure 4 clusters with size in the interval [10,19], and Figure 5 clusters with size < 10. Given the large number of tissue samples in the data, this categorization of the cluster sizes facilitates extraction of features from the images. An interesting characteristic of the clustering results is that several classes of tissue samples emerge, such that the members have amplifications in more than a single chromosome (see in particular Figures 4 and 5). This finding motivates further the treatment of all chromosomal bands simultaneously within the model, instead of splitting the cluster analyses over the distinct chromosomes. The analysis with k-means using 291 clusters identified a clustering which was relatively similar to the model-based clustering, with adjusted Rand-Index [28] between the two partitions being 0.659. However, although k-means discovered mostly coherent clusters, some of the identified clusters also contained samples which had few features in common. For example, the largest cluster identified in the k-means analysis seems to contain samples which are more or less randomly assigned together. The fifteen largest clusters from the k-means analysis are graphically presented in Figure 6.

**Figure 7:**
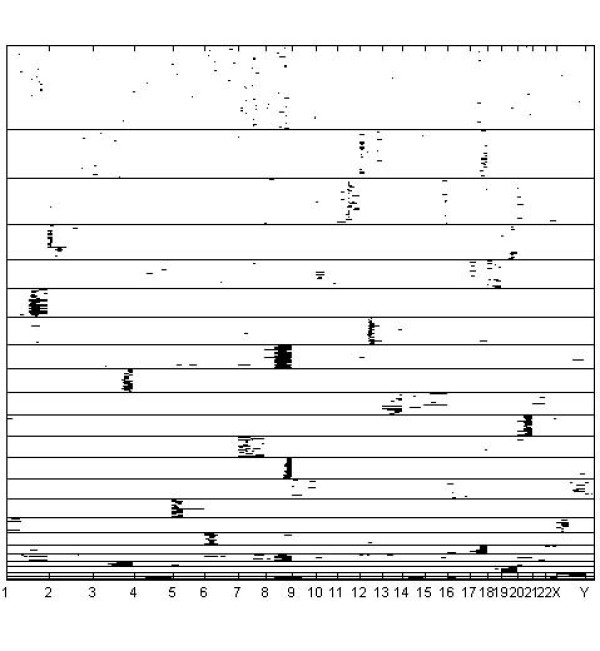
**Results with the basic beta-binomial model for the cancer data**. Results for the DNA copy number amplification data, when the basic beta-binomial model was used in the analysis.

**Figure 8:**
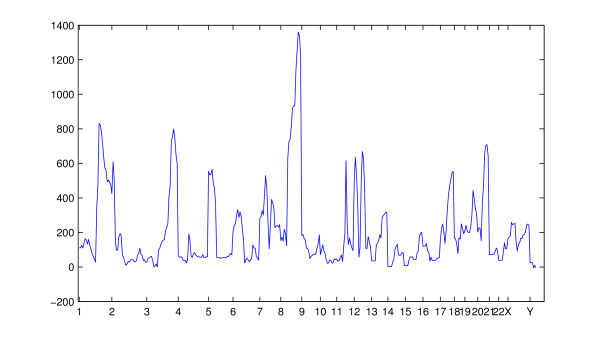
**Log posterior odds for the informativeness of features**. Profile of logarithm of posterior odds for the sub-bands in the DNA copy number amplification data being informative against them being uniformative. The sub-bands are shown on x-axis, and the y-axis shows the corresponding log posterior odds values.

When the copy number amplification data were analyzed using the simple beta-binomial model with BAPS software, the algorithm converged to a solution with 26 clusters only. These results are presented graphically in Figure 7. A highly similar clustering result was obtained with AutoClass, which yielded 32 clusters. The adjusted Rand Index between these two results was .40, which is a high value given the large size of the data base. We abstain from presenting the AutoClass results visually, as the clustering image is nearly identical to Figure 7. The observed inability of a standard mixture model without feature selection to detect majority of the biologically interesting patterns is in harmony with the corresponding results from the analyses of synthetic data reported above. In Figure 8, we illustrate the behavior of the log posterior odds for the sub-bands being informative against them being uniformative, over the chromosomes. The relatively high information contents of certain chromosomal areas compared to others is clearly visible in the curve.

The Bayesian clustering approach allowed us to determine cancer subtypes that were associated with a specific DNA copy number amplification pattern. A cross-reference table between the obtained clustering and the known cancer types as well as the details of the clustering, including the amplification patterns for the clusters, are presented in the Additional files 2 and 3. The results illustrate the fact that subtypes of same cancer type may differ in their genetic make-up. Identified amplification patterns represent specific subtypes of given cancer types. For example, five main subtypes of colorectal adenocarcinoma are characterized by amplification patterns of 13q11-13q34 (11.4%), 20q11.1-20q13.3 (8.6%), 20q13.2 (6.4%), 8q11.1-8q24.3 (5.7%) and 20p13-20q13.3 (5.7%). These subtypes may have different clinical behaviour and treatment requirements.

There is a new category of drugs that target receptor tyrosine kinases (RTKs) [29,30]. RTKs are membrane proteins that are involved in signal transduction and are often implicated in cancer. In addition to mutations and protein over-expression, gene amplification is one of the main mechanisms that incur the oncogenic activity of the RTKs. Novel small molecule or antibody drugs have been developed to treat cancers that manifest ill-activated RTKs and inhibit the enzymatic activity of the kinase domain or receptor binding of these proteins. There are approved cancer therapies that target receptor tyrosine kinases: Trastuzumab inhibits *ERBB2 *(17q12), Imatinib targets *ABL *(9q34.1), *KIT *(4q12) and *PDGFR *(5q31-q32), Gefitinib and Cetuximab interact with *EGFR *(7p12.3-p12.1), and Bevecizumab hinders *VEGF *(6p12) activity [31]. In addition to the approved use in treatment of metastatic breast cancer (Trastuzumab), *CML *and *GIST *(Imatinib), non-small cell lung cancer (Gefitinib) and colorectal cancer (Cetuximab and Bevecizumab) [29] these tyrosine kinase targeting drugs could be used to treat other malignancies that have amplifications of the specific drug target genes.

Generally, the Bayesian method yielded similar results as the previous probabilistic modeling study [2], where data from distinct chromosomes were treated separately. Figures 3, 4, 5 suggest that this is due to the relatively low level of noise in these data. However, our experiments with the standard mixture modeling approach similar to that used by [2], show that a completely different pattern emerges when the joint database over all chromosomes is considered, leading to very poor resolution under the standard model. Clear advantage in using the Bayesian approach is that the genome-wide modeling method facilitated the identification of amplification patterns, which encompassed areas from multiple chromosomes. There were 12 amplification patters that formed clusters of 2 to 16 cases: 1q and 3q [n = 16]; 5p14-p12 and 8q11-8q24.3 [n = 13]; 17q and 20q12-q13.3 [n = 9]; 1q and 6p [n = 7]; 1q and 8q [n = 7]; 12q24 and 20q12-q13 [n = 6]; 3q26-q29 and 7q21-q35 [n = 6]; 19q11-q13 and 20q11-q13.3 [n = 5]; 7 and 9 [n = 4]; 1q, 16p and 19q [n = 4]; 11q and Xq21-q28 [n = 4]; 16p, 20p, 21p and 22q [n = 2]. Because most of the multichromosomal amplification patterns encompassed large genomic regions and only few local amplification sites were identified, the site specificity and mechanisms of multichromosomal amplifications remains unclear. q-arm of chromosome 1 and 20q12-q13 regions seemed to be frequently present in the disjoined amplification patterns.

Multichromosomal amplification patterns seemed to be randomly distributed among the cancer types and were not associated with any group of specific malignancies. The few numbers of cases in the clusters did not allow further inferences about the mechanisms or cancer type specificity of multichromosomal DNA copy number amplifications.

## Conclusion

In this work, we applied a Bayesian model-learning approach to sub-classify human cancers based on DNA copy number amplifications. An inherent part of the model structure was the division of the considered chromosomal sub-bands into informative and noninformative subsets, and further the specification of group-specific amplifications for each detected cluster. Thus, both the clustering and the interesting amplification patterns are immediately available from our approach. Another advantage offered by the Bayesian paradigm is that it constitutes a firm basis for inferring the number of distint components (i.e. clusters) in the model. Furthermore, the results illustrate the importance of considering jointly all the chromosomes, as several clusters having group-specific amplifications in different chromosomes were identified.

Rather than looking at the appearance or behavior of a tumor, categorizing cancers according to their underlying genetic properties is likely to improve cancer management. Tumors that arise in the same part of the body may appear histologically similar but still have totally different molecular changes. Conversely, cancers from different origins and morphology could be addicted to same disturbed genes and protein pathways. Understanding the genetic underpinnings of cancers and reclassification in molecular-level will help to develop new cancer treatments and apply currently available treatments to seemingly unrelated cancers.

The presented analyses illustrate the potential residing in machine learning and pattern recognition approaches as a tool for discovering biologically vital information from large databases, even when the measurements are relatively noisy. However, they also highlight the usefulness of tailoring the data mining methods to take into account particular biological features of the available measurements. In contrast, blind use of standard methods ignoring such features may yield poor insights about the underlying biological reality.

## Authors' contributions

PM and JC developed the Bayesian modeling approach and the algorithms. PM implemented the learning methods. PM and JC carried out the analyses. SM participated in the design of the study and evaluated the results for the cancer data. All authors contributed to the writing of the final manuscript.

## Supplementary Material

Additional file 1 **Table 1S.** Results of validatory experiments with learning from synthetic data. Click here for file

Additional file 2 **Clusters and cancer types.** A cross-reference table between the obtained clustering and the known cancer types for the DNA copy number amplification data. Click here for file

Additional file 3 **Clustering details.** Details of the clustering results for the DNA copy number amplification data, including the amplification patterns for different clusters Click here for file

## References

[B1] Myllykangas S, Himberg J, Böhling T, Nagy B, Hollmén J, Knuutila S (2006). DNA copy number amplification profiling of human neoplasms. Oncogene.

[B2] Myllykangas S, Tikka J, Böhling T, Knuutila S, Hollmén J (2008). Classification of human cancers based on DNA copy number amplification modeling. BMC Medical Genomics.

[B3] Mitelman F, Johansson B, Mertens F (1994). Catalog of Chromosome Aberrations in Cancer.

[B4] Myllykangas S, Böhling T, Knuutila S (2007). Specificity, selection and significance of gene amplifications in cancer. Seminars in Cancer Biology.

[B5] Bock K (1996). Language production: Methods and methodologies. Psychonomic Bulletin & Review.

[B6] Jain AK, Duin RPW, Mao J (2000). Statistical Pattern Recognition: A Review. IEEE Transactions on Pattern Analysis and Machine Intelligence.

[B7] Tikka J, Hollmén J, Myllykangas S, Sandoval F, Prieto A, Cabestany J, Graña M (2007). Mixture modeling of DNA copy number amplification patterns in cancer. Proceedings of the 9th International Work-Conference on Artificial Neural Networks.

[B8] Robert C, Casella (2005). Monte Carlo Statistical Methods.

[B9] Geyer CJ, Thompson EA (1995). Annealing Markov Chain Monte Carlo with Applications to Ancestral Inference. Journal of American Statistical Association.

[B10] Jensen ST, Liu XS, Zhou Q, Liu JS (2004). Computational Discovery of Gene Regulatory Binding Motifs: A Bayesian Perspective. Statistical Science.

[B11] Marttinen P, Corander J, Törönen P, Holm L (2006). Bayesian search of functionally divergent protein subgroups and their function specific residues. Bioinformatics.

[B12] Corander J, Gyllenberg M, Koski T (2007). Random partition models and exchangeability for Bayesian identification of population structure. Bulletin of Mathematical Biology.

[B13] Bernardo JS, Smith AFM (1994). Bayesian Theory.

[B14] Corander J, Gyllenberg M, Koski T (2006). Bayesian model learning based on a parallel MCMC strategy. Statistics and Computing.

[B15] Corander J, Marttinen P, Mäntyniemi S (2006). Bayesian identification of stock mixtures from molecular marker data. Fishery Bulletin.

[B16] Ripley BD (1996). Pattern Recognition and Neural Networks.

[B17] Kass R, Raftery AE (1995). Bayes factors. Journal of American Statistical Association.

[B18] Dy JG, Brodley CE (2004). Feature selection for unsupervised learning. Journal of Machine Learning Research.

[B19] Law MHC, Figueiredo MAT, Jain AK (2004). Simultaneous feature selection and clustering using mixture models. IEEE Transactions on Pattern Analysis and Machine Intelligence.

[B20] Cheeseman P, Stutz J, Fayyad U, Piatetsky-Shapiro G, Smyth P, Uthurusamy R (1996). Bayesian classification (AutoClass): Theory and results. Advances in Knowledge Discovery and Data Mining.

[B21] Gyllenberg M, Koski T, Verlaan M (1997). Classification of binary vectors by stochastic complexity. Journal of Multivariate Analysis.

[B22] Peña JM, Lozano JA, Larrañaga P (2002). Learning recursive Bayesian multinets for data clustering by means of constructive induction. Machine Learning.

[B23] Zhang NL (2004). Hierarchical latent class models for cluster analysis. Journal of Machine Learning Research.

[B24] Santafé G, Lozano JA, Larrañaga P (2006). Bayesian model averaging of naive Bayes for clustering. IEEE Transactions on Systems, Man, and Cybernetics-Part B:Cybernetics.

[B25] Corander J, Marttinen P (2006). Bayesian identification of admixture events using multi-locus molecular markers. Molecular ecology.

[B26] Gelman A, Carlin JB, Stern HS, Rubin DB (2004). Bayesian Data Analysis.

[B27] Neal RM (2000). Markov Chain Sampling Methods for Dirichlet Process Mixture Models. Journal of Computational and Graphical Statistics.

[B28] Hubert L, Arabie P (1985). Comparing partitions. Journal of Classification.

[B29] Gschwind A, Fischer OM, Ullrich A (2004). The discovery of receptor tyrosine kinases: targets for cancer therapy. Nature Reviews Cancer.

[B30] Imai K, Takaoka A (2006). Comparing antibody and small-molecule therapies for cancer. Nature Reviews Cancer.

[B31] Baselga J (2006). Targeting tyrosine kinases in cancer: the second wave. Science.

