# Supplementary material for: Bayesian clustering and feature selection for cancer tissue samples
Source: BMC Bioinformatics. 2009 Mar 18;10:90. doi: 10.1186/1471-2105-10-90 (PMC2679022; doi:10.1186/1471-2105-10-90)
Supplement: Additional file 1 — Table 1S. Results of validatory experiments with learning from synthetic data. [file 1471-2105-10-90-S1.pdf]

Table 1S. Results of validatory experiments with learning from synthetic data

(a) $k = 30$:

.01				.05				.10			
$\alpha = .875$	$\alpha = .90$	$\alpha = .95$	$\alpha = .975$	$\alpha = .875$	$\alpha = .90$	$\alpha = .95$	$\alpha = .975$	$\alpha = .875$	$\alpha = .90$	$\alpha = .95$	$\alpha = .975$
.292	.344	.439	.467	.242	.278	.402	.420	.215	.266	.334	.373
13.60	14.80	17.35	18.60	11.60	13.45	16.55	17.90	11.01	12.70	15.60	17.25
.360	.390	.427	.432	.278	.339	.384	.384	.268	.308	.372	.378
.435	.436	.410	.383	.407	.421	.399	.369	.398	.411	.404	.373
$\alpha = .875$	$\alpha = .90$	$\alpha = .95$	$\alpha = .975$	$\alpha = .875$	$\alpha = .90$	$\alpha = .95$	$\alpha = .975$	$\alpha = .875$	$\alpha = .90$	$\alpha = .95$	$\alpha = .975$
.708	.765	.891	.919	.651	.707	.869	.880	.722	.778	.916	.941
22.45	23.70	27.00	28.05	21.85	23.15	26.50	27.20	23.10	24.60	27.75	28.45
.622	.655	.718	.716	.612	.649	.707	.684	.652	.691	.741	.713
.431	.403	.304	.261	.424	.403	.312	.284	.408	.372	.280	.256
$\alpha = .875$	$\alpha = .90$	$\alpha = .95$	$\alpha = .975$	$\alpha = .875$	$\alpha = .90$	$\alpha = .95$	$\alpha = .975$	$\alpha = .875$	$\alpha = .90$	$\alpha = .95$	$\alpha = .975$
.946	.963	.992	.993	.936	.978	.989	.994	.971	.980	.989	.995
28.45	28.80	29.70	29.70	28.15	29.35	29.70	29.90	28.95	29.30	29.70	29.85
.878	.878	.879	.829	.862	.900	.873	.837	.896	.900	.876	.832
.210	.200	.158	.176	.226	.159	.151	.158	.199	.179	.170	.176

(b) $k = 50$:

.01				.05				.10			
$\alpha = .875$	$\alpha = .90$	$\alpha = .95$	$\alpha = .975$	$\alpha = .875$	$\alpha = .90$	$\alpha = .95$	$\alpha = .975$	$\alpha = .875$	$\alpha = .90$	$\alpha = .95$	$\alpha = .975$
.988	.989	.992	.993	.973	.956	.973	.981	.924	.940	.967	.970
48.05	48.25	48.65	48.80	45.60	46.20	47.25	48.10	45.50	46.15	47.55	48.25
.901	.903	.894	.875	.841	.846	.841	.827	.835	.843	.836	.814
.234	.222	.205	.205	.296	.285	.261	.244	.294	.275	.248	.236
$\alpha = .875$	$\alpha = .90$	$\alpha = .95$	$\alpha = .975$	$\alpha = .875$	$\alpha = .90$	$\alpha = .95$	$\alpha = .975$	$\alpha = .875$	$\alpha = .90$	$\alpha = .95$	$\alpha = .975$
.999	.999	.999	.998	.999	.999	.999	.999	.999	.999	.999	.999
50.00	50.00	50.10	50.15	50.00	50.00	50.00	50.10	50.00	50.00	50.00	50.00
.978	.976	.956	.936	.988	.984	.966	.950	.989	.986	.974	.959
.058	.059	.084	.101	.041	.050	.077	.091	.036	.042	.064	.081
$\alpha = .875$	$\alpha = .90$	$\alpha = .95$	$\alpha = .975$	$\alpha = .875$	$\alpha = .90$	$\alpha = .95$	$\alpha = .975$	$\alpha = .875$	$\alpha = .90$	$\alpha = .95$	$\alpha = .975$
1.000	1.000	1.000	1.000	.999	1.000	1.000	.999	.999	.999	.999	.999
50.00	50.00	50.00	50.00	50.00	50.00	50.00	50.00	50.00	50.00	50.00	50.15
.999	.997	.993	.986	.998	.997	.992	.986	.995	.993	.986	.974
.006	.014	.029	.044	.009	.017	.031	.045	.022	.030	.048	.063

The values .01, .05, .1 refer to the defined levels of noise in the generating model. The three rows of each table panel (a,b) correspond to the generating distributions of the cluster sizes: Uniform(5,20), Uniform(10,25), Uniform(15,30), in the given order (top-down). Each column of

four values under a specific value of α reports means over 20 simulated data sets generated using the same design (k, distribution of the number of clusters, noise level) and analyzed using the proposed method. The four reported values are (top-down): adjusted Rand Index, number of clusters in the optimal partition, mean percentage (over clusters) of the group-specific amplifications correctly discovered by the optimal model structure, std.dev. of the percentage (over clusters) of the group-specific amplifications correctly discovered by the optimal model structure.
